# Peptide-Conjugated Vascular Endothelial Extracellular Vesicles Encapsulating Vinorelbine for Lung Cancer Targeted Therapeutics

**DOI:** 10.3390/nano14201669

**Published:** 2024-10-17

**Authors:** Isha Gaurav, Abhimanyu Thakur, Kui Zhang, Sudha Thakur, Xin Hu, Zhijie Xu, Gaurav Kumar, Ravindran Jaganathan, Ashok Iyaswamy, Min Li, Ge Zhang, Zhijun Yang

**Affiliations:** 1 School of Chinese Medicine, Hong Kong Baptist University, Hong Kong SAR 999077, China; ishagaurav.botany@gmail.com (I.G.);; 2 Department of Pharmacology, Delhi Pharmaceutical Sciences & Research University (DPSRU), New Delhi 110017, India; 3 Department of Neurosurgery, Massachusetts General Hospital, Harvard Medical School, Boston, MA 02115, USA; 4 Ben May Department for Cancer Research, Pritzker School of Molecular Engineering, University of Chicago, Chicago, IL 60637, USA; 5 National Institute for Locomotor Disabilities (Divyangjan), Kolkata 700090, India; 6 State Key Laboratory of Resource Insects, Medical Research Institute, Southwest University, Chongqing 400715, China; 7 Department of Pathology, Xiangya Hospital, Central South University, Changsha 410017, China; 8 National Clinical Research Center for Geriatric Disorders, Xiangya Hospital, Central South University, Changsha 410017, China; 9 Clinical Research Division, Department of Biosciences, School of Basic and Applied Sciences, Galgotias University, Greater Noida 203201, India; 10 Preclinical Department, Universiti Kuala Lumpur, Royal College of Medicine Perak (UniKL-RCMP), Ipoh 30450, Malaysia; 11 Mr. & Mrs. Ko Chi-Ming Centre for Parkinson’s Disease Research, School of Chinese Medicine, Hong Kong Baptist University, Hong Kong SAR 999077, China; 12 Department of Biochemistry, Karpagam Academy of Higher Education, Coimbatore 641021, India; 13 Law Sau Fai Institute for Advancing Translational Medicine in Bone and Joint Diseases, School of Chinese Medicine, Hong Kong Baptist University, Hong Kong SAR 999077, China; 14 Institute of Integrated Bioinfomedicine and Translational Science, School of Chinese Medicine, Hong Kong Baptist University, Hong Kong SAR 999077, China; 15 Institute of Precision Medicine and Innovative Drug Discovery, HKBU Institute for Research and Continuing Education, Shenzhen 518000, China

**Keywords:** lung cancer, drug delivery, extracellular vesicles, exosomes, HUVEC, GE11 peptide, vinorelbine, targeted delivery

## Abstract

Lung cancer is one of the major cancer types and poses challenges in its treatment, including lack of specificity and harm to healthy cells. Nanoparticle-based drug delivery systems (NDDSs) show promise in overcoming these challenges. While conventional NDDSs have drawbacks, such as immune response and capture by the reticuloendothelial system (RES), extracellular vesicles (EVs) present a potential solution. EVs, which are naturally released from cells, can evade the RES without surface modification and with minimal toxicity to healthy cells. This makes them a promising candidate for developing a lung-cancer-targeting drug delivery system. EVs isolated from vascular endothelial cells, such as human umbilical endothelial-cell-derived EVs (HUVEC-EVs), have shown anti-angiogenic activity in a lung cancer mouse model; therefore, in this study, HUVEC-EVs were chosen as a carrier for drug delivery. To achieve lung-cancer-specific targeting, HUVEC-EVs were engineered to be decorated with GE11 peptides (GE11-HUVEC-EVs) via a postinsertional technique to target the epidermal growth factor receptor (EGFR) that is overexpressed on the surface of lung cancer cells. The GE11-HUVEC-EVs were loaded with vinorelbine (GE11-HUVEC-EVs-Vin), and then characterized and evaluated in in vitro and in vivo lung cancer models. Further, we examined the binding affinity of ABCB1, encoding P-glycoprotein, which plays a crucial role in chemoresistance via the efflux of the drug. Our results indicate that GE11-HUVEC-EVs-Vin effectively showed tumoricidal effects against cell and mouse models of lung cancer.

## 1. Introduction

Cancer is a major health problem all over the world [1,2,3,4]. According to the Global Burden of Disease, cancer claimed approximately 10 million people’s lives in 2020 [5]. There are more than a hundred types of cancer that affect different organs or tissues, including lung, breast, and brain. Among various types, lung cancer is the most common and has a relatively high mortality rate [6,7,8,9]. Despite extensive research, patients with lung cancer have poor prognoses, as 50% of patients die within a year of diagnosis and the average 5-year survival rate is less than 18% [10]. Lung cancer can be broadly classified into the following two groups: small cell lung cancer (SCLC) and non-small cell lung cancer (NSCLC); they account for approximately 20% and 80% of all lung cancers, respectively [11]. NSCLC can be further grouped into the following three types: squamous-cell carcinoma, large-cell carcinoma, and adenocarcinoma. Generally, lung cancer can be treated based on its type and how far it has spread. The major treatment options for NSCLC include surgery, radiation therapy, chemotherapy, or their combination, whereas for SCLC only radiation therapy and chemotherapy are used [12]. However, there are various limitations related to the abovementioned treatment options. For example, surgery may not lead to a long-term result, and there is a chance of relapse [13]; radiotherapy can damage healthy tissues surrounding the tumor [14]; and chemotherapy has limitations associated with solubility, selectivity, toxicity, and development of resistance to anticancer drugs [15]. Therefore, there is a demand for the development of a targeted therapy with high bioavailability and minimum toxicity for both types of lung cancer.

Nanotechnology has played an important role in cancer therapeutics because drug delivery via the encapsulation of anticancer agents in nanocarriers is efficient [16,17,18]. Recently, nanoparticles (NPs), such as liposomes, polymer-based formulations, and metal-based NPs (MNPs), have been designed for delivering anticancer drugs and nucleic acids such as DNA and siRNA to metastatic lung cancer cells efficiently [19,20,21,22]. Although, NP-based drug delivery systems (NDDSs) have several advantages, such as reduced toxicity and enhanced bioavailability [23], artificially synthesized NDDSs such as liposomes and MNPs can evoke an immune response and become engulfed by the reticuloendothelial system (RES) [24]. We hypothesize that using extracellular vesicles (EVs) for delivering drugs to cancer cells can overcome these difficulties. EVs are membranous nanovesicles secreted by most cell types, such as cancer cells, endothelial cells, and neuron cells, and these secreted EVs circulate in most biofluids such as blood and pleural fluid. Depending on how they are generated and their size, EVs are differentiated into the following three types: exosomes, microvesicles (MVs), and apoptotic bodies (ABs). Exosomes are the smallest EVs, with diameters of 30–150 nm [25]. EVs including exosomes are capable of cell-to-cell communication and are used by the body to deliver diverse payloads, such as proteins, lipids, and nuclear acids, to recipient cells [26,27]. EVs are created when inward budding in the plasma membrane results in the formation of intermediate endosome vesicles and multivesicular bodies (MVBs). MVBs either fuse with lysosomes and degrade or fuse with the plasma membrane and form exosomes that are eventually released from cells into the extracellular milieu [21,28]. MVs are 500 nm–2 µm in diameter. They are released via budding off from the cell membrane [22]. ABs are the largest, at 50–5000 nm in diameter. They are released by cells undergoing apoptosis [29]. These size ranges are approximate, and debate persists, as many of them overlap [30]. Specific proteins are present on the surface of exosomes. These proteins are unique for the endosomal pathways that characterize the exosome and differentiate it from MVs and ABs. Such proteins include tetraspanin proteins (CD9, CD63, and CD81) [31], lysosomal protein (HSP70), tumor-susceptibility gene 101 (TSG101), and fusion proteins (annexin and flotillin) [32]. Interestingly, EVs have already been widely studied for their application in drug delivery. EVs loaded with bioactive cargoes of miRNA and protein have been found to function by delivering these cargoes to tumor cells [33,34]. In addition, EVs can also inhibit tumors by delivering chemical drugs [35]. Importantly, EVs, as natural carriers of chemical drugs, can avoid phagocytosis by macrophages and prolong the half-life of chemical drugs compared to delivery carriers [36]. Consequently, in the process of delivery, EVs can significantly improve the efficiency of delivering certain biodegradable drugs [37,38]. Notably, the engineering of EVs via surface modification has shown great potential as a therapy, and engineered EVs play key roles in treating many conditions, such as cancer, inflammation, and neurological diseases [39,40,41].

Human umbilical endothelial cells (HUVECs) have shown anti-angiogenic activity in a lung cancer mouse model [42]. In addition, the mice immunized with the endothelial-cell-based vaccine showed reduced human esophageal squamous cell carcinoma (ESCC) development and increased T-lymphocyte recruitment in the spleen [43]. Another study showed that HUVECs induced by a tumor microenvironment (TME) using the supernatant of murine CT26 colorectal cancer cells exerted a better antiangiogenic effect than the HUVECs themselves [44]. EVs isolated from HUVEC-derived EVs (HUVEC-EVs) could be potential carriers for drug delivery. Further, as the epidermal growth factor receptor (EGFR) is overexpressed on the surface of different types of cancer cells in the TME including lung cancer cells [45], we proposed to engineer HUVEC-EVs via GE11 peptide, which binds specifically to EGFR and has been widely utilized for targeted drug delivery [46]. Thereby, we proposed to develop GE11-HUVEC-EVs by postinserting GE11 peptide into HUVEC-EVs.

Reports suggest that weekly vinorelbine monotherapy may be a feasible therapeutic option for patients with heavily treated, advanced NSCLC, particularly lung adenocarcinoma [47,48]. Another study demonstrated that vinorelbine improves the survival of elderly patients with advanced NSCLC and possibly enhances overall quality of life [49]. However, the adverse effect, owing to the toxicity of vinorelbine, is still a challenge. Therefore, encapsulating vinorelbine in EVs would facilitate a reduction in its toxicity and improve its safety profile, thereby minimizing its adverse effect [50]. We believe the GE11-HUVEC-EVs loaded with vinorelbine (GE11-HUVEC-EVs-Vin) could be an effective EV-based drug delivery system (EDDS) for lung cancer therapy. This led us to propose to load the GE11-HUVEC-EVs with vinorelbine, followed by their characterization, and evaluation in in vitro and in vivo lung cancer models.

P-glycoprotein (P-gp) plays a significant role in chemoresistance by actively pumping out a wide range of chemotherapeutic drugs from cancer cells, thereby reducing their intracellular concentrations and effectiveness. This efflux activity of P-gp prevents a sufficient accumulation of anticancer drugs within the cell, allowing cancer cells to avoid the cytotoxic or apoptotic effects of these drugs [51]. Therefore, we also examined the binding affinity between ABCB1 protein (which encodes for P-gp) and vinorelbine, as well as compared with controls such as elacridar, azithromycin, clarithromycin, and erythromycin.

## 2. Materials and Methods

### 2.1. Ethics Statement

Animal-related experiments were conducted as per protocols approved by the Committee on the Use of Human and Animal Subjects in Teaching and Research (HASC) at the Hong Kong Baptist University (HKBU) (#REC/21-22/0265).

### 2.2. Analysis of Single-Cell and Bulk RNA-Seq Data

The single-cell RNA sequencing dataset GSE127465 [52] was obtained from a public database, the Gene Expression Omnibus (GEO). The dataset features the total cells (n = 40,362) in the biopsies of patients with lung tumors (n = 7). The single-cell portal singlecell.broadinstitute.org (Study# SCP739, accessed on 20 October 2023) was utilized for generating two-dimensional visualizations (SPRING plots) and the dot-plot showing the cell-type-specific gene expression.

Lung-cancer-specific bulk-RNA seq data were analyzed to obtain the expression level of EGFR in pan-cancer (https://tnmplot.com/analysis/ (accessed on 21 November 2022) [53]), and the *EGFR* expression-based survival plots were generated using https://kmplot.com/analysis/ (accessed on 21 November 2022) [54]. TNMplot includes 56,938 unique samples from the GEO, GTex, TCGA, and TARGET databases. This includes 15,648 normal, 40,442 tumor, and 848 metastasis samples [53]. For kmplot, gene expression and survival data were sourced from GEO, EGA, and TCGA. A PostgreSQL server manages the database, integrating gene expression with clinical data concurrently [54].

For the analysis of the immunotherapeutic response, the data from patients were classified into responders and nonresponders based on the pathological response or survival duration. Responders had a progression-free survival of over 12 months or showed a partial or complete response. Nonresponders had a PFS of under 12 months or exhibited progressive or stable disease [55].

### 2.3. In Silico Analysis for Target Identification

Lung cancer targets were collected through online databases, such as GeneCards (https://www.genecards.org/), OMIM (https://www.omim.org/search/advanced/geneMap), Therapeutic Target Database (https://db.idrblab.net/ttd/), and PharmMapper (http://www.lilab-ecust.cn/pharmmapper/results/240204030418.html), among others. The potential targets of compounds were predicted using SuperPred (https://sea.bkslab.org/) and SEA (https://sea.bkslab.org/). A total of 74 small-molecule targets and 1520 lung cancer targets were collected, with an intersection yielding 24 targets. These targets were imported and analyzed and visualized using Cytoscape_v3.9.0. The network consisted of 24 nodes and 110 edges.

### 2.4. Molecular Docking

The 3D atomic coordinates for the crystal structures of Nanodisc-reconstituted human ABCB1 in a complex with MRK16 Fab and elacridar (PDB id: 7A6C) were downloaded from the RCSB-Protein Data Bank (PDB). Additionally, the 3D structures of azithromycin, clarithromycin, erythromycin, elacridar, and vinorelbine were retrieved from the PubChem database. The structure of ABCB1 was prepared for molecular docking by loading it into UCSF Chimera.

A grid box was generated to encompass the elacridar-binding site of ABCB1 and provide ample space for the ligands’ rotational and translational movements. The grid box parameters were set as follows: the center grid box coordinates were set to 164.622, 159.652, and 157.799 for the X, Y, and Z dimensions, respectively, with a spacing of 0.375 Å and the number of points set to 84, 96, and 86 points in the X, Y, and Z dimensions.

AutoDock Tools 1.5.6 was utilized to perform molecular docking using the Lamarckian genetic algorithm’s (LGA) search parameters. Subsequently, LigPlot+ (v.1.4.5) was employed to visualize the interaction patterns between the ABCB1 and ligand in the complexes.

### 2.5. Cell Culture

HUVECs and A549 cells were cultured in a standard medium containing Dulbecco’s Modified Eagle Medium (DMEM) (Cat. #11965084, Gibco, Waltham, MA, USA) and supplemented with 10% fetal bovine serum (FBS) (Cat. #10500064, Gibco), and 1% penicillin–streptomycin solution. The cultured cells were maintained at 37 °C in an incubator with 5% CO_2_.

### 2.6. Isolation of HUVEC-EVs and GE11-HUVEC-EVs

EVs, including exosomes, were extracted from the HUVEC cell culture medium using the Total Exosome Isolation (TEI) reagent (Thermo Scientific, Waltham, MA, USA, Cat. #4478359) [56]. The process involved collecting the medium, centrifuging it at 2000× *g* for 30 min, mixing the supernatant with TEI reagent, and incubating it at 4 °C overnight. Subsequently, the mixture was centrifuged at 10,000× *g* for 1 h at 4 °C. The resulting pellet, which contained EVs, was then diluted with 1× phosphate-buffered saline (PBS) for analysis.

### 2.7. Surface Engineering of HUVECs to Express GE11 Peptide (GE11-HUVEC-EVs)

The modification of HUVECs to express GE11 peptide was accomplished using the postinsertion technique. In brief, the GE11 peptide was dissolved in 4-(2-hydroxyethyl)-1-piperazineethanesulfonic acid buffer for 15 min at 60 °C to form micelles. Then, the HUVEC-EV suspension was mixed with the above suspension for 2 h at 40 °C. After cooling to room temperature, EVs were immediately purified by size-exclusion chromatography to obtain GE11-modified HUVEC EVs (GE11-HUVEC-EVs). 

### 2.8. Analysis of the Sizes and Concentration of EVs via Nano Tracking Analyzer

The size distribution and concentration of EVs were assessed using a Malvern Nanoparticle Tracking Analysis (NTA) system. A 405 nm laser beam was employed to analyze 500 mL of EV solutions in the sample chamber. NTA software (version 2.2, NanoSight NS300, Salisbury, UK) was utilized to analyze the captured videos of EVs in Brownian motion, allowing for the determination of the size distribution and concentration of EVs.

### 2.9. Transmission Electron Microscopy (TEM)

The sizes, shapes, and morphologies of the EVs were characterized using transmission electron microscopy (TEM). A negative staining technique was employed, where a 30 μL drop in the EV suspension in filtered PBS was placed on carbon-coated electron microscope grids, incubated at room temperature for 10 min, transferred to a drop of Uranyless^®^ solution for 1 min, and air dried; the excess stain was blotted away, and the grids were observed using a TEM machine.

### 2.10. Immunogold EM Analysis

The presence of CD63 protein on EVs was confirmed using immunogold-EM as described previously [56] Initially, EV suspensions, fixed with 2% PFA, were adsorbed to CCEM grids and washed in a solution of 0.05 M filtered glycine in PBS. Subsequently, the grids were incubated with anti-CD63 primary antibody (Abcam, Cambridge, UK, Cat. #ab68418) for 24 h at 4 °C, followed by incubation with 10 nm gold-conjugated donkey anti-rabbit antibody (Abcam, Cat. #ab27234) for 1 h at room temperature. After several washes, the grids were post-fixed, excess fluid was blotted, and the samples were dried before imaging using TEM machine (FEI Spirit 120 kV LaB6 Electron Microscope, Hillsboro, OR, USA).

### 2.11. Imaging Flow Cytometry Analysis of EVs

The cells were stained with anti-EGFR-conjugated Alexa 488 antibody (Abcam, Cat. #ab193244), followed by analysis with image flow cytometry as described previously with slight modification [57]. Briefly, the cells were immune-stained with anti-EGFR-conjugated primary antibody for 1 h at room temperature, followed by evaluation of the cell samples with an imaging flow cytometer equipped with a laser. The images were acquired and analyzed using IDEAS software version 6.2 [58].

### 2.12. Fourier-Transform Infrared (FT-IR) Microscopy

The EV samples with and without peptide functionalization were analyzed with FT-IR via the potassium bromide (KBr) technique. Briefly, the EV samples were diluted (ratio = 1:10) in potassium bromide, followed by making pellets using a mini hand-held laboratory hydraulic press. A KBr pellet alone was used as a control. The FTIR instrument was used to obtain signals from the pellets of the EV samples with and without peptide functionalization, as well as the KBr alone.

### 2.13. Loading of Vinorelbine on HUVEC-EVs and GE11-HUVEC-EVs and Their Evaluation

EVs were loaded with vinorelbine using sonication. Equal amounts of vinorelbine and EVs were mixed and sonicated using 20% amplitude and 6 cycles of 30 s on/150 s off. Subsequently, the solution was incubated at 37 °C for 60 min. To separate the excess free drug, size-exclusion chromatography with a Sephadex G25 column (Cytiva, Uppsala, Sweden) was employed. The efficiency of drug loading was assessed using a UV-visible spectrophotometer.

### 2.14. Labeling of EVs

EVs were labeled with Exo-Green Fluorescent Labeling reagent (Cat. #EXOG200A-1, System Biosciences, Palo Alto, CA, USA). A total of 50 µL of 10× Exo-Green was added to a 500 µL EV solution in 1XPBS (200 µg protein) and mixed by flicking. The solution was then incubated for 10 min at 37 °C, and the labeling reaction was stopped by adding FBS. After incubation at 4 °C for 30 min, the solution was centrifuged at 14,000 rpm for 3 min to remove excess label. The labeled EV pellet was then resuspended in PBS for subsequent monitoring.

### 2.15. EV Uptake Assay

Recipient A549 cells were cultured at a density of 30,000 cells per well on Lab-Tek chamber slides (Thermo Scientific, Waltham, MA, USA) for 24 h. After 24 h, the cells were washed with PBS and then exposed to a medium supplemented with 50 μg/mL Exo-Green labeled- HUVEC-EVs and GE11-HUVEC-EVs. The concentration of EVs for the uptake assay was chosen based on previously published literature [59]. Subsequently, the recipient cells were washed with PBS, fixed with 4% PFA on ice for 30 min, and then washed again with PBS. The cells were stained, and the slide was covered with a thin layer of Vectashield medium containing DAPI for visualization under a confocal microscope at 40× magnification.

### 2.16. Cell Viability Assay

The MTT cell viability assay was conducted on A549 cells (10,000 cells per well) in a 96-well microtiter tissue culture plate. After 48 h, the cells were treated with HUVEC-EVs, GE11-HUVEC-EVs, vinorelbine (Vin), HUVEC-EVs-Vin, or GE11-HUVEC-EVs-Vino for 24 h. Following treatment, an MTT solution was added to each well, and the cells were incubated for 4 h at 37 °C. Subsequently, DMSO was added to solubilize the formazan product, and the absorbance was measured at 570 nm.

### 2.17. Migration Assay

The migration of the A549 cells (control or with various treatments) was evaluated via the scratch- as well as Transwell- migration assay. The scratch assay involves the creation of a “scratch” in a cell monolayer, typically using a pipette tip, to simulate a wound. The cells were then incubated, and images were captured at the beginning and at regular intervals during cell migration to close the scratch. The rate of migration was quantified by comparing the images, and the time required to close the wound.

For Transwell assay, A549 cells (1 × 10^5^ cells/100 μL of medium per well) were seeded in Transwell chambers with 8.0 μm pores (Costar, Washington, DC, USA, Cat. #3422). The upper chamber contained cells with or without treatments, while the lower chamber held the culture medium. After 24 h of migration, cells in the lower chamber were fixed, stained with Crystal Violet, and quantified using microscopy and ImageJ software version v1.54.

### 2.18. Apoptosis Assay

The level of apoptosis in the A549 cells (control and with various treatments) was evaluated via the Annexin-V staining of cells. The staining process involved incubation of the cells with the Annexin V antibody, which could bind to phosphatidylserine (PS) on the surface of apoptotic cells. The annexin-V-stained cells were examined via fluorescent microscopy.

### 2.19. Immunocytochemistry

The immunocytochemistry procedure was conducted on A549 cells grown on poly-D-lysine-coated glass cover slips until they reached 70% confluence. The cells were then treated with Exo-Green-labeled EV samples, washed with PBS, fixed with 4% paraformaldehyde, and permeabilized with 0.1% Triton X-100 in PBS. Subsequently, the cells were blocked with 1% BSA in PBS. Rhodamine-phalloidin-conjugated primary antibody solution was used, and samples were mounted in a Vectashield medium with DAPI. The samples were examined using a confocal microscope (Leica TCS SP8, Weitzlar, Germany).

### 2.20. Development of Cancer Mouse Model

A lung cancer cell-based mouse model was developed to study the antitumor effects of GE11-HUVEC-EVs-Vin in vivo. This model was established using the implantation technique. Briefly, the A549 cell suspension was prepared in Matrigel Matrix (BD Biosciences, Franklin Lakes, NJ, USA), which is commonly used as an anchor to prevent tumor cells from spreading at the site of injection. SCID mice (6–8 weeks) were anesthetized and placed in the right lateral decubitus position. One milliliter syringes with 30-gauge needles were used to inject A549 cells percutaneously into the left lateral thorax, at the lateral dorsal axillary line, about 1.5 cm above the lower rib line, just below the inferior border of the scapula. After tumor cell injection, the labeled EV samples, namely, HUVEC-EVs, GE11-HUVEC-EVs, HUVEC-EVs-Vino, and GE11-HUVEC-EVs-Vino, and Vinorelbine, were injected intravenously (4 µg of EVs/mouse). The mice were observed until they fully recovered. In the 9th week after tumor cell implantation, mice were sacrificed, and lung tumors were collected. Tumor formation was confirmed via H&E staining, and EGFR expression was confirmed in the tumors using immunohistochemistry.

### 2.21. Hematoxylin–Eosin (H&E) Staining

H&E staining was carried out on xenograft tumors, fixed with 4% paraformaldehyde (PFA), embedded in paraffin, and sectioned into 5 μm slices using an ultra-thin semiautomatic microtome. The sections were then deparaffinized, rehydrated, and stained with hematoxylin and eosin (H&E) using standard protocols. The H&E-stained sections were examined under a light microscope.

### 2.22. Immunohistochemistry

The lungs of mice (control and with various treatments) were processed for immunohistochemistry by preparing PFA-fixed, frozen, and cryomatrix-embedded sections. These sections were then subjected to immunostaining analysis using primary antibodies for EGFR and Ki67, along with EXO-Green-labeled EVs. Following the staining, the sections were imaged, and images were analyzed using Image J analysis (NIH) software version v1.54. This comprehensive process allowed for the examination of protein expression and EV localization in specific lung regions.

### 2.23. Statistical Analysis

Differences were statistically evaluated using a one-way analysis of variance (ANOVA), followed by Fisher’s protected least significant difference test. A *p*-value < 0.05 was considered statistically significant.

## 3. Results

### 3.1. EGFR Gene Overexpression Is Associated with Poor Survival of Patients with Lung Cancer

**Figure 1:**
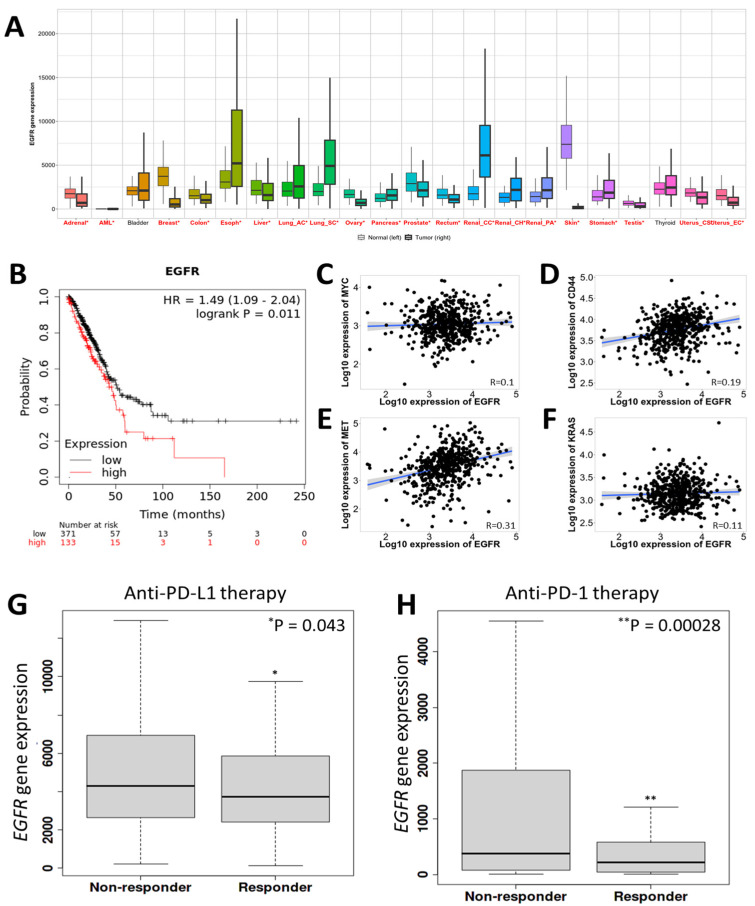
High expression of EGFR is correlated with poor survival of patients with lung cancer. (**A**) Bar graphs showing the expression of EGFR in different types of cancer compared to their respective controls. The red-colored cancer type depicts a significant difference between the normal and tumor groups. (**B**) Survival curve showing the time dependent probability of survival with EGFR expression in patients with lung cancer. (**C**–**F**) Graphs showing the positive correlations between (C) EGFR and MYC, (**D**) EGFR and CD44, (**E**) EGFR and MET, and (**F**) EGFR and KRAS. (**G**,**H**) Bar graphs showing the expression of EGFR in the (**G**) nonresponder (N = 269) and responder (N = 185) groups toward the treatment of anti-PD-L1 therapy and (**H**) nonresponder (N = 277) and responder (N = 166) groups toward the treatment of anti-PD-1 therapy. The difference between the nonresponders and responders was compared using Mann–Whitney test. Significance level set at * *p* < 0.05 and ** *p* < 0.01.

The EGFR gene is important in cancer because it plays a role in many of the key processes that drive cancer development and progression, including cell growth, division, survival, and migration. EGFR overexpression can lead to cancer by making cancer cells more resistant to apoptosis (programmed cell death) and more likely to metastasize [60]. Therefore, it is pertinent to examine the correlation of the EGFR gene with the overall survival of patients with lung cancer. Notably, the pan-cancer analysis using RNA-seq data from the TCGA database showed that EGFR is, in general, overexpressed in most cancers including lung cancer compared to their respective control groups (Figure 1A). We also found that a high expression of EGFR is correlated with the poor survival of patients with lung cancer (Figure 1B). Moreover, the lung cancer stage-based analysis showed that in stage IV, median survival is far less while the expression of EGFR is heightened (Supplementary Figure S1A–D). A single-cell transcriptomic analysis of data from patients with lung cancer (GEO: GSE127465) [52] revealed cell-type-specific distribution of EGFR in the lung cancer TME (Supplementary Figure S2A–E). Further, EGFR expression was positively correlated with MYC, CD44, MET, and KRAS, although it is noteworthy that the correlations of EGFR with MYC and KRAS are very weak, and only the correlation of EGFR with MET is somewhat stronger (Figure 1C–F). MYC is a transcription factor that regulates the expression of many genes involved in cell growth, division, and metabolism. MYC overexpression is common in many types of cancer, including lung cancer [61]. CD44 is a cell surface protein that helps cells interact with their environment. CD44 overexpression has been linked to cancer cell stemness, invasion, and metastasis [62]. MET is a receptor tyrosine kinase that is activated by hepatocyte growth factor (HGF). MET overexpression can lead to cancer cell proliferation, survival, and migration [63]. KRAS is a small GTPase that plays a role in cell signaling. KRAS mutations are common in many types of cancer, including lung cancer, colon cancer, and pancreatic cancer. The expression of EGFR is also linked with major markers, contributing to lung cancer development and progression (Figure 1C–F).

Programmed death-ligand 1 (PD-L1) and programmed death-1 (PD-1) are two proteins that play a key role in lung cancer immune evasion. PD-L1 is expressed on the surface of cancer cells and other cells in the TME. PD-1 is expressed on the surface of T cells, which are a type of white blood cell that plays a key role in the immune response. When PD-L1 interacts with PD-1, it sends a signal to the T cell to stop it from attacking the cancer cell. This allows the cancer cell to evade the immune system and continue to grow and spread. PD-L1 overexpression is common in lung cancer, and it is associated with a poor prognosis. Patients with PD-L1-overexpressing tumors are more likely to have shorter overall survival and are less likely to respond to immunotherapy [64]. Immunotherapy is a type of cancer treatment that harnesses the body’s immune system to fight cancer. Immune checkpoint inhibitors are a type of immunotherapy that blocks the interaction between PD-L1 and PD-1. This allows T cells to recognize and attack cancer cells [65]. Immune checkpoint inhibitors are effective in treating some patients with lung cancer, especially those with PD-L1-overexpressing tumors. Targeting the PD-L1/PD-1 pathway with immunotherapy is a promising approach to cancer treatment [64,65]. Therefore, we further analyzed the effect of anti-PD-L1 or anti-PD-1 therapy on the EGFR via bioinformatics analysis using the TCGA dataset. Interestingly, the expression of EGFR was significantly reduced in the responder group toward both anti-PD-L1 and anti-PD-1 therapy (Figure 1G,H). We conclude that EGFR is a crucial target for developing targeted therapeutics for cancers including lung cancer.

### 3.2. EGFR Protein Is Significantly Overexpressed in the A549 Cells in Hypoxic TMEs

**Figure 2:**
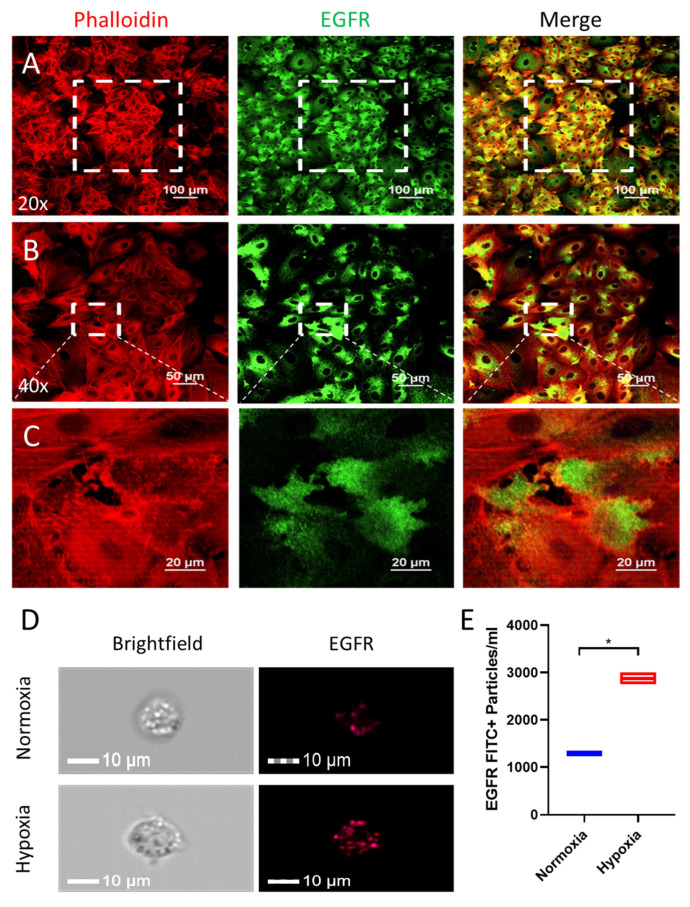
Enhanced expression of EGFR on lung cancer cells under hypoxia. Immunofluorescence microscopy showing the expression and distribution pattern of EGFR protein (green color) in A549 lung cancer cells at (**A**,**B**) 20× and 40× magnifications and (**C**) the corresponding enlarged images. (**D**,**E**) Image flow cytometry-based expression of EGFR on the A549 cells under normal and hypoxic conditions (24 h incubation) and the corresponding quantitative bar graph. Comparison between the normoxia and hypoxia groups was performed using the student’s *t*-test with a significance level of * *p* < 0.01.

As overexpression of EGFR on the surface of lung cancer cells has been linked with their malignant behavior [45,66], we anticipated that a ligand that targets EGFR could be used for lung cancer therapeutics if it were conjugated on the surface of EVs. We performed immunocytochemistry (ICC), which helps to reveal the distribution of EGFR on the surface of lung cancer cells using anti-EGFR primary antibodies. Interestingly, the ICC results show that EGFR is primarily localized on the surface of A549 lung cancer cells (Figure 2A–C). As hypoxia, or low oxygen levels, is a common feature of the TME and is closely associated with cell proliferation, angiogenesis, metabolism, and tumor immune response, it can promote tumor progression, increase its aggressiveness, and enhance the metastatic potential—all of which translates to a poor prognosis [67]. Therefore, we examined the effect of hypoxia (1% O_2_ level) on the protein level of EGFR in A549 cells via image flow cytometry [58]. The level of EGFR was significantly enhanced in the hypoxia-exposed A549 cells compared to the control A549 cells (Figure 2D,E). In addition, the level of EGFR was also found to be highly upregulated in lung cancer cell lines (A549 and HCC2814) compared to the HEK293 cell line (Supplementary Figure S2F). These results show that EGFR would be a good target for the delivery of medicine in lung cancer treatment.

### 3.3. Endothelial-Cell-Derived EVs Attenuate the Migration of Lung Cancer Cells

**Figure 3:**
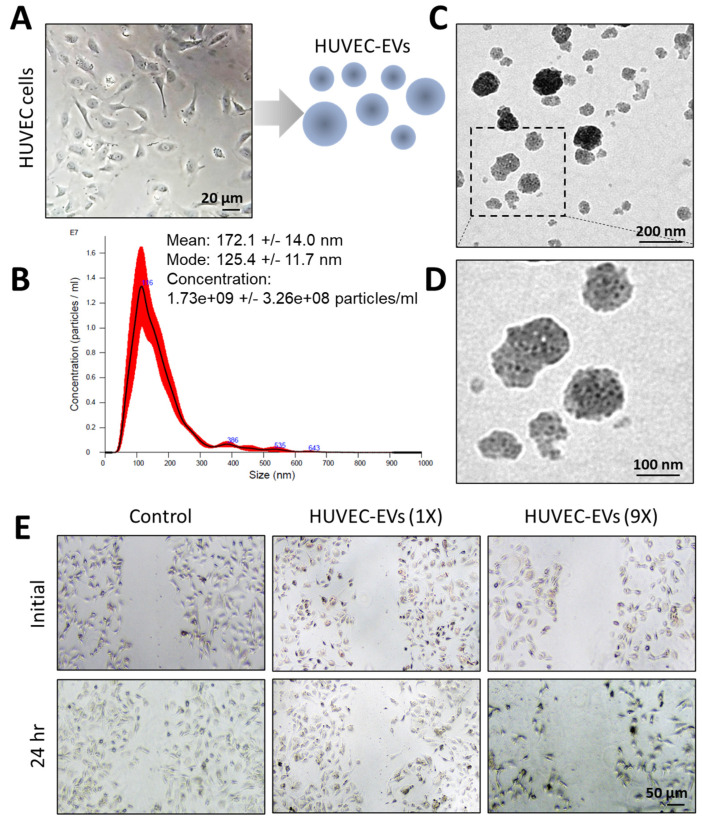
Isolation and characterization of EVs from endothelial cells: (**A**) representative bright-field image of HUVECs that had been cultured in EV-depleted medium for 24 h, before the isolation of EVs. (**B**–**D**) Representative (**B**) size distribution plot of the HUVEC-EVs; (**C**,**D**) immunogold dots showing the expression of CD63 on the HUVEC-EVs. (**E**) Representative brightfield images showing the migration of A549 cells at the start and 24 h. after the addition of HUVEC-EVs at different dilutions (1× and 9× with x = 1.73 × 10^9^ particles/mL). Scale bars = (**A**), 20 nm; (**C**,**D**), 100 nm; (**E**), 50 nm.

Vascular endothelial cell-derived EVs have been found to inhibit the malignant progression of lung cancer [42], suggesting that endothelial cells could be an apt source of EVs for developing EV-based drug delivery tools. HUVEC cells were first cultured in a complete medium, then in an EV-depleted medium. Figure 3A shows a brightfield image of a typical HUVEC cell, which was utilized for EV isolation. Subsequently, the HUVEC-EVs were characterized with a nano-tracking analyzer and electron microscope. The average diameter of HUVEC-EVs was found to be around 172 nm, and the concentration of released EVs was 1.73 × 10^9^ particles/mL (Figure 3B). The size distribution and zeta potential analysis showed an insignificant difference between GE11-HUVEC-EVs and GE11-HUVEC-EVs-Vin, shown in Supplementary Figure S3A–C. Electron microscopy showed the morphology of the EVs, and immunogold labeling revealed the expression of CD63, an EV biomarker (Figure 3C,D). This further established that the isolated vesicles were indeed EVs. The EVs were added to the culture of A549 lung cancer cells at a concentration of 50 µg/mL and incubated at room temperature for 24 h. Notably, the HUVEC-EVs were found to inhibit the migration of lung cancer cells (Figure 3E), demonstrating that they are a suitable source of EVs for lung cancer therapeutics.

### 3.4. Engineering Endothelial Cells EVs via GE11 Peptide Postinsertion and Loading of Vinorelbine

**Figure 4:**
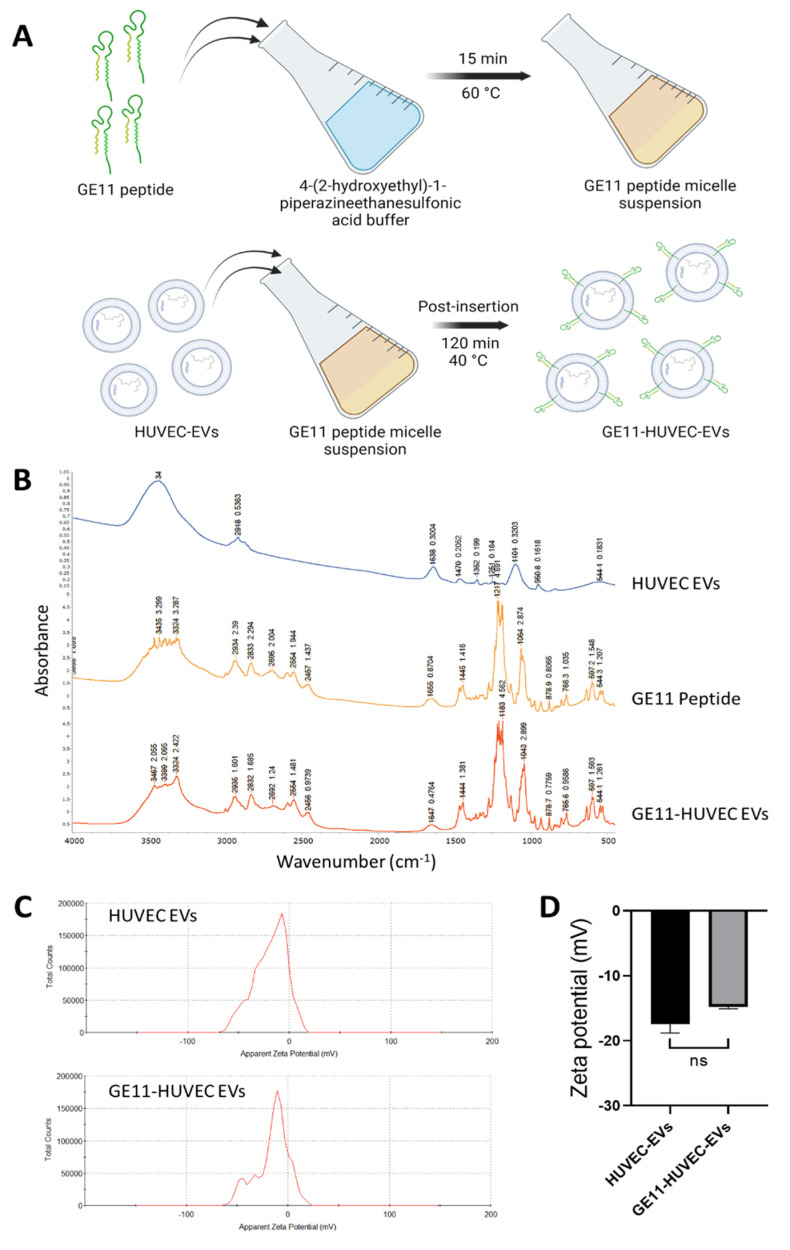
Characterization of engineered EVs with GE11 peptide via postinsertion technique: (**A**) a schematic showing the functionalization of HUVEC-EVs with the postinsertion of GE11 peptide; (**B**) representative FT-IR graphs with peaks characteristic for HUVEC-EVs, GE11 peptide, and GE11-HUVEC-EVs; (**C**,**D**) zeta potential graphs and the corresponding quantitative bar graph for the EVs before and after postinsertion of the GE11 peptide. Data are shown as the mean ± S.E.M. (N = 2). The statistical analysis was performed using the Student’s *t*-test for the control HUVEC-EVs vs. GE11-HUVEC-EVs. Significance levels set at * *p* < 0.05 and ** *p* < 0.01; ns = not significant.

As we found that HUVEC-EVs could be useful for inhibiting the malignant phenotype of lung cancer cells, we further questioned whether we could tailor the EVs to achieve the specific targeting of lung cancer cells. As most of the cancer cells including lung cancer cells express high levels of EGFR [66], we engineered the HUVEC-EVs via postinsertion of GE11 peptide, which has been reported to interact with the EGFR receptor [45,46,68]. Briefly, the GE11 peptide was dissolved in 4-(2-hydroxyethyl)-1-piperazineethanesulfonic acid buffer for 15 min at 60 °C, during which time micelles formed. This was then mixed with HUVEC-derived EVs suspension and incubated for 2 h at 40 °C. Next, after cooling to room temperature, EVs were immediately purified by size-exclusion chromatography to obtain GE11-modified HUVEC-EVs (GE11-HUVEC-EVs) (Figure 4A). The engineered EVs were analyzed by FT-IR to confirm the successful insertion of GE11 peptide into the EVs. Figure 4B shows the FT-IR-based graphs and marked characteristic peaks for the HUVEC-EVs, GE11 peptide, and GE11-HUVEC-EVs. The characteristic FT-IR peaks of the GE11-HUVEC-EVs were found to be like those of the GE11 peptide and included the peaks of HUVEC-EVs, suggesting successful and efficient functionalization of HUVEC-EVs with GE11 peptide. Based on the present data, a part of the GE11 peptide might either be on the surface or inside an EV. Size distribution analysis revealed a small shift in size after the conjugation with GE11 peptide. There was a slight change in the value of the zeta potential of the HUVEC-EVs after the functionalization with a GE11 peptide, but it was not very significant (Figure 4C,D). This suggests that the postinsertion of the GE11 peptide into the HUVEC-EVs was accomplished without much impact on the properties of the EV membranes.

We identified ABCB1 as a potential target for lung cancer via in silico target identification analysis (Supplementary Figure S4A–D), as described in Section 2. The *ABCB1* gene, which codes for P-glycoprotein, plays crucial physiological roles, particularly in the protection of cells and organs against toxic compounds. Owing to its ability to recognize and transport a broad range of substrates, this transporter can impart a chemoresistance phenotype to cancer cells. Elacridar has been found to have a potent effect on docetaxel-resistant NSCLC cells [68]. We examined the binding affinity of vinorelbine toward the ABCB1 protein and compared it with those of elacridar and other relevant drugs. Azithromycin, clarithromycin, and erythromycin were used as additional controls. Notably, it was found that the binding energy for the interaction between vinorelbine and ABCB1 was comparable to that between elacridar and ABCB1 (Supplementary Figure S5 and Table S1). Therefore, we concluded that vinorelbine would be an appropriate candidate anticancer drug for loading into engineered EVs. The GE11-HUVEC-EVs were loaded with vinorelbine (GE11-HUVEC-EVs-Vin) via a sonication method, and the UV-visible spectroscopy analysis showed that the loading efficiency was approximately 32.67% (Supplementary Figure S6 and Table S2). We used GE11-HUVEC-EVs-Vino to examine its anticancer effect against lung cancer cells in vitro and in vivo.

### 3.5. GE11-Peptide-Engineered EVs Were Incorporated into EGFR-Expressing Lung Cancer Cells and Showed Tumoricidal Effects In Vitro

**Figure 5:**
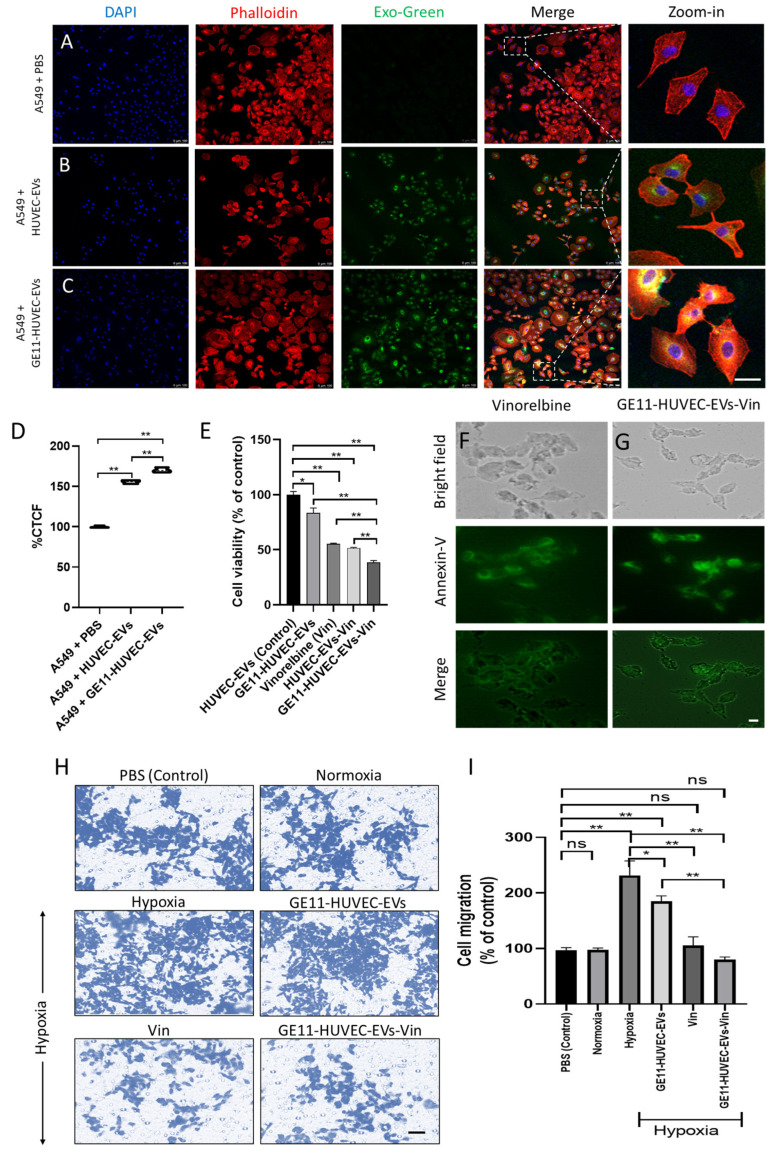
Effect of GE11-HUVEC-EVs-Vin on malignant phenotypes of A549 lung cancer cells. (**A**–**D**) GE11-peptide-engineered endothelial cell EVs are efficiently internalized by lung cancer cells. Representative (**A**–**C**) immunofluorescence images and (**D**) a violin plot showing the uptake of (**A**) PBS, (**B**) HUVEC-EVs, and (**C**) GE11-HUVEC-EVs by the A549 cells (scale bar = 100 nm). (**E**) GE11-HUVEC-EVs-Vin significantly reduced the cell viability of A549 cells. Representative bar graph showing the effect of the following different treatment groups—HUVEC-EVs, GE11-HUVEC-EVs, vinorelbine (Vin), HUVEC-EVs-Vin, and GE11-HUVEC-EVs-Vino—on the proliferation of A549 cells, as detected by the MTT cell viability assay. (**F**,**G**) Representative immunofluorescence images showing the expression of Annexin-V in A549 cells treated with Vin or HUVEC-EVs-Vin (scale bar = 100 nm). (**H**,**I**) GE11-HUVEC-EVs significantly reduced the migration of A549 cells under hypoxia. (**H**) Representative images from the Transwell chamber (scale bar = 100 nm) and (**I**) a bar graph showing the effects of the following different treatment groups—HUVEC-EVs, GE11-HUVEC-EVs, Vinorelbine, HUVEC-EVs-Vin, and GE11-HUVEC-EVs-Vino—on the migration ability of A549 cells, as detected by the Transwell migration assay under hypoxia, compared to the vehicle-treated and untreated normoxia. Data are shown as the means ± standard error means (S.E.M.), with N = 3 replicates per group. Significance levels set at * *p* < 0.05 and ** *p* < 0.01; ns = not significant. Statistical comparisons were performed for the control vs. HUVEC-EVs; control vs. GE11-HUVEC-EVs; control vs. vinorelbine; control vs. HUVEC-EVs-Vin; and control vs. GE11-HUVEC-EVs-Vin with one-way ANOVA. For comparison of the HUVEC-EVs or GE11-HUVEC-EVs or vinorelbine or HUVEC-EVs-Vin with GE11-HUVEC-EVs-Vin, the Student’s *t*-test was applied.

To examine the effectiveness of the GE11-HUVEC-EVs, an uptake assay was performed by incubating A549 cells with PBS, HUVEC-EVs, or GE11-HUVEC-EVs. The EV samples were labeled with EXO-Green, whereas the A549 cells’ membranes and nuclei were stained with phalloidin and DAPI, respectively. Interestingly, the confocal analysis revealed that the GE11-HUVEC-EVs were significantly taken up by the A549 cells but not the HUVEC-EVs, as evident from the significant co-localization of Exo-Green and phalloidin in A549 cells treated with labeled EVs (Figure 5A–D). Further, the GE11-HUVEC-EVs loaded with vinorelbine (GE11-HUVEC-EVs-Vino) showed a significant effect on the cell viability as evident from the decline in number of A549 cells (Figure 5E). Supplementary Figure S7A–E show that GE11-HUVEC-EVs-Vino had a relatively less effect on the migration, as well as the proliferation, of normal HEK293 cells compared to A549 cells, which could be attributed to the basal expression of EGFR in HEK293 cells (Supplementary Figure S2F). The enhanced level of Annexin-V, an apoptotic marker (Figure 5F,G), and the decline in the migration ability of the cells treated with GE11-HUVEC-EVs-Vin under hypoxia compared to vehicle-treated and untreated normoxia (Figure 5H,I) also suggest the potential of GE11-HUVEC-EVs as an effective therapeutic for lung cancer.

### 3.6. GE11-HUVEC-EVs-Vin Showed a Tumoricidal Effect in an In Vivo Lung-Cancer-Cell-Based Tumor Mouse Model

**Figure 6:**
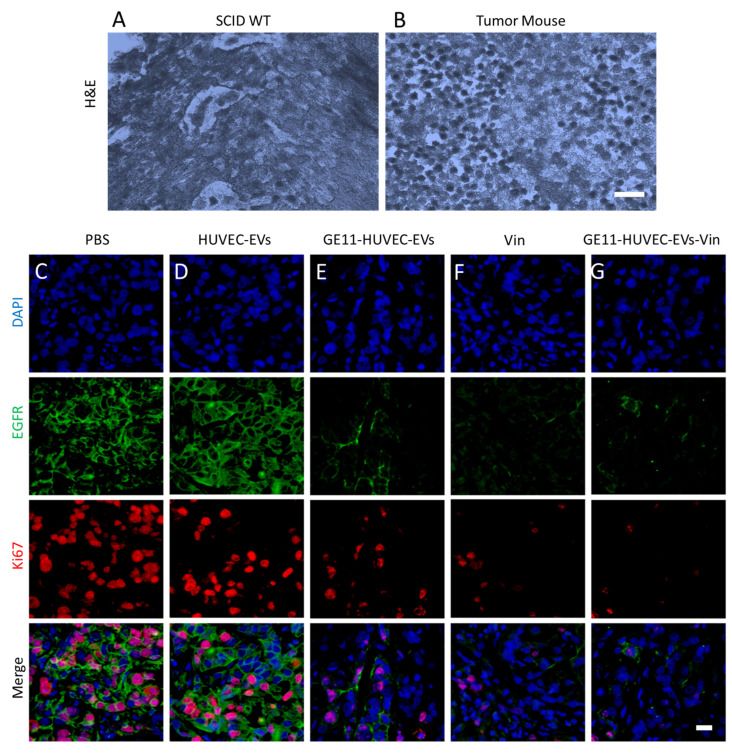
GE11-HUVEC-EVs-Vin significantly reduced the expression of EGFR and Ki67 in the tumor tissue of a mouse model of lung cancer: (**A**,**B**) representative H&E staining of the lung tissue of SCID WT and lung-cancer-cell-based tumor mouse model (scale bar = 50 µm); (**C**–**G**) representative immunofluorescence images showing the effects of different treatments—HUVEC-EVs, GE11-HUVEC-EVs, vinorelbine (Vin), HUVEC-EVs-Vin, and GE11-HUVEC-EVs-Vin—on nuclei (depicted by blue color; DAPI), EGFR (green), and Ki67 (red) in a lung cancer cell-based tumor mouse model (scale bar = 50 µm).

To validate the in vitro results in vivo, we developed a xenograft model of lung cancer via injection of A549 cells into SCID mice, as per the protocol (approval number: REC/21-22/0265) of the Research Ethics Committee at Hong Kong Baptist University. The H&E staining of tumor tissue samples showed significant malignant cell proliferation (as evident from the high number of stained nuclei) compared to the control mice, which established the formation of the tumor mice model (Figure 6A,B). Fixed tissue sections from the tumor mice model treated with PBS, HUVEC-EVs, GE11-HUVEC-EVs, vinorelbine (Vin), and GE11-HUVEC-EVs-Vin were stained with DAPI, EGFR, and Ki67 (a marker for proliferating cells). Notably, the expression levels of EGFR and Ki67 were significantly reduced in the mice treated with GE11-HUVEC-EVs-Vin; the levels were comparable with the effect of vinorelbine alone (Figure 6C–G, Supplementary Figure S8A). The H&E staining of different organs in the lung cancer mouse model showed that the engineered EVs did not exhibit major toxicity in vivo (Supplementary Figure S8B–G). This suggests that GE11-HUVEC-EVs-Vin are effective as a lung cancer therapeutic in vivo.

## 4. Discussion

EGFR overexpression is a common feature of lung cancer and is associated with poor patient survival. It has been found that EGFR overexpression drives cancer cell proliferation, migration, and invasion and also confers resistance to conventional therapy. Even with our in silico bioinformatics analysis using the TCGA dataset, we found that EGFR is highly expressed in lung cancer cells, and the high expression of EGFR is correlated with the poor survival of patients with lung cancer (Figure 1A–H and Figure 2A–C). Therefore, developing ways to target EGFR overexpression appears to be a potentially effective yet previously unexplored strategy for treating lung cancer, which is what we aimed to do in this study.

HUVECs have shown anti-angiogenic activity in lung cancer mouse models [42]. This compelled us to examine the anti-migration ability of HUVEC-EVs, as autologous EVs have been reported to mimic the constituents, as well as the phenotypes, of their parent cells [69,70]. Because of this, we isolated EVs from HUVECs and incubated them with A549 lung cancer cells. It was found that the HUVEC-EVs inhibited the migration of lung cancer cells (Figure 3A–E), demonstrating their aptness as a source of EVs for lung cancer therapeutics.

EV-based targeted delivery is of paramount importance in cancer therapy. EVs, with their small size, biocompatibility, and low immunogenicity, are ideal drug carriers. They can deliver various therapeutic agents, such as drugs, nucleic acids, and proteins, to cancer cells, enhancing drug stability and achieving tissue-specific targeted delivery. Tumor-derived EVs exhibit a remarkable ability to target cancer cells, offering a promising approach for effective, personalized cancer treatment. Genetically engineered EVs loaded with anticancer drugs hold even more potential [71,72,73]. The peptide-based functionalization of EVs has also attracted attention owing to their ability to accomplish targeted delivery [74,75]. Particularly, GE11, a synthetic peptide that binds to EGFR with high specificity and affinity, is effective for targeting cancer cells in vitro and in vivo [46]. In this study, we, for the first time, decorated HUVEC-EVs with GE11 peptides via postinsertion (Figure 4A). Postinsertion offers several advantages for targeted drug delivery in cancer therapy. Unlike genetic modification at the cellular level, postinsertion allows for the introduction of targeting moieties in EVs after their isolation. This method is relatively simple and avoids gene-related side effects, providing a more controlled and specific approach to functionalizing EVs. Additionally, it facilitates the induction of functionality in lipid membrane-based nanomaterials without the limitations of direct chemical modification methods [76,77]. Here, our studies confirms the potential of this method. Evaluation of EVs with FT-IR and DLS established their successful engineering with the GE11 peptide (Figure 4B), without much impact on their membrane potential (Figure 4C).

Using autologous EVs for drug delivery to the same origin of parent cells offers significant advantages. These EVs, derived from the patient’s cells, are well-tolerated and less likely to trigger immune responses. They also reflect the phenotypic state of the parent cells, enhancing their specificity and effectiveness in delivering therapeutic agents to the target cells. This personalized approach holds great potential for improved treatment outcomes in cancer therapy [69,78]. As we chose endothelial-cell-derived EVs, it was imperative that HUVEC-EVs were attracted to the endothelial cells, which are crucial components of the TME [79]. Further, we examined the uptake of GE11-HUVEC-EVs by A549 cells, and, importantly, GE11-HUVEC-EVs were internalized by the A549 cells relatively better than HUVEC-EVs, suggesting the successful ability of GE11 to target EGFR (Figure 5A–C). Subsequently, the GE11-HUVEC-EVs-Vin were found to be effective in showing tumoricidal effect toward the lung cancer cells in vitro (Figure 5D–H) as well as in vivo (Figure 6A–G). The engineered GE11-HUVEC-EVs-Vin showed relatively less effect on the migration and proliferation of the normal HEK293 cells, which depicts the relative specificity of our GE11-engineered EV-based delivery approach, as EGFR is highly expressed in A549 cancer cells compared to normal HEK293 cells. While GE11 targeting is significant, similar cellular responses suggest the need to elucidate the specific advantages of EV-mediated Vin delivery. Potential benefits could include improved drug stability, targeted delivery to specific tissues, or enhanced cellular uptake. Further investigation into pharmacokinetics and off-target effects would clarify the EVs’ therapeutic value in this context. Conclusively, the engineered endothelial-cell-derived EVs with GE11 peptide via postinsertion method were found to be an effective targeted drug delivery approach for potential lung cancer therapeutics.

Despite the effectiveness of our strategy in in vitro studies and a pre-clinical model, several challenges persist. The first challenge is dealing with the fact that tumor cells also release EVs. Tumor-derived EVs can promote tumor growth and metastasis [80,81]. Therefore, it is important to develop means to distinguish between engineered EVs and tumor-derived EVs. Another problem is that engineered EVs may be degraded by enzymes in the bloodstream or taken up by nontarget cells. Therefore, it is important to engineer EVs to make them resistant to degradation and to target them specifically to tumor cells [80]. A third challenge is obtaining the appropriate quantity of peptide-functionalized EVs. Endosomal proteases in the cell can break down peptides during EV production. To stop peptide degradation, Huang et al. conjugated a targeting peptide with a glycosylation sequence using a genetic engineering technique [82].

In the future, the safety and efficacy of GE11-peptide-engineered EVs in patients with lung cancer must be evaluated. If proven successful in practice, this EV-engineering-based targeted drug delivery strategy could be extended to other types of cancer by customizing the EVs with different ligands against cancer-specific receptors. Further, the source of EVs can be changed depending on the cell type that is being targeted.

## Data Availability

All the data and relevant supporting data have been presented in the manuscript. For additional information or data, the corresponding author can be asked with a reasonable request.

## Supplementary Materials

The following supporting information can be downloaded at: https://www.mdpi.com/article/10.3390/nano14201669/s1, Supplementary Figure S1. Stage-dependent survival of patients with lung cancer and with the corresponding expression level of *EGFR*. Survival curves of patients with lung cancer with the expression of *EGFR* in (A) stage I, (B) stage II, (C) stage III, and (D) stage IV. Supplementary Figure S2. Single-cell RNA-seq analysis of the TME in patients with lung cancer: (A) UMAP depicting the different cell types in the TME of human non-small cell lung cancer compared to the control group (based on GEO: GSE127465); (B,C) UMAPs showing the expressions of (B) *EGFR* and (C) *ERBB2* in the clusters, representing cancer cell types; (D,E) Dot plots showing the cell-type-specific expression of major cancer markers including *EGFR* and *ERBB2*. The figures are based on the analysis of single-cell RNA-seq data publicly available (GSE127465); (F) expression levels of EGFR in HEK293, A549, and HCC2814 cell lines, as analyzed using protein atlas. Supplementary Figure S3: Size distribution of GE11-HUVEC-EVs. The graph shows the size-dependent concentration of GE11-HUVEC-EVs. Supplementary Figure S4: Identification of potential lung cancer targets via in silico analysis: (A) the intersection of compound target proteins and lung cancer treatment targets resulted in 44 intersecting targets; (B) these 24 potential targets were imported into the STRING (https://cn.string-db.org/) online website to obtain a PPI network, which contains 24 nodes and 110 edges. The depth of color and circle size represent the corresponding size of the degree; (C) GO enrichment analysis was conducted on these 24 potential targets, and the top 10 enrichment results are displayed; (D) KEGG enrichment analysis was performed on these 24 potential targets, and the top 30 enrichment results are displayed. Supplementary Figure S5: Molecular docking of vinorelbine with ABCB1 protein. The H-bonding and hydrophobic interaction patterns of the control drugs (azithromycin, clarithromycin, erythromycin, and elacridar) and vinorelbine with ABCB1 are depicted. Supplementary Figure S6: Evaluation of vinorelbine encapsulation in GE11-HUVEC-EVs: (A) UV-visible spectra of the difference in the concentrations of vinorelbine standard solutions; (B) calibration standard graph showing the linear regression between the concentration of vinorelbine standard solutions and the corresponding absorbance at a wavelength of 269 nm (λ_max_). Supplementary Figure S7: Effect of engineered GE11-HUVEC-EVs-Vin on the migration of HEK293 cells. Representative brightfield images of initial and 24 h after the various treatments (as indicated in images) to the HEK293 cells. Supplementary Figure S8: Evaluation of the in vivo toxicity of GE11-HUVEC-EVs in major organs of mice: (A) bar graph showing the fluorescence intensity depicting the expression levels of EGFR and Ki67; (B–G) H&E-stained images of major organs from lung cancer xenograft mice: (B) brain, (C) heart, (D) lung, (E) liver, (F) spleen, and (G) kidney. Table S1: Docking of different ligands with ABCB1. Table S2: Calculation of the efficiency of vinorelbine encapsulation in GE11-HUVEC-EVs.
