# Supplementary material for: Peptide-Conjugated Vascular Endothelial Extracellular Vesicles Encapsulating Vinorelbine for Lung Cancer Targeted Therapeutics
Source: Nanomaterials (Basel). 2024 Oct 17;14(20):1669. doi: 10.3390/nano14201669 (PMC11510406; doi:10.3390/nano14201669)
Supplement: Supplementary file 1 [file nanomaterials-14-01669-s001.zip › nanomaterials-3167382-supplementary.pdf]

SUPPLEMENTARY DATA

Peptide-Conjugated Vascular Endothelial Extracellular Vesicles Encapsulating Vinorelbine for Lung Cancer Targeted Therapeutics

Isha Gaurav[1], Abhimanyu Thakur[2,3], Kui Zhang[4], Sudha Thakur[5], Xin Hu[6], Zhijie Xu[7,8], Gaurav Kumar[9], Ravindran Jaganathan[10], Ashok Iyaswamy[1,11,12], Min Li[1,11], Ge Zhang[13,14,15], Zhijun Yang[1*]

[1]School of Chinese Medicine, Hong Kong Baptist University, Hong Kong SAR 999077, China.
[2]Department of Pharmacology, Delhi Pharmaceutical Sciences & Research University (DPSRU), New Delhi 110017, India.
[3]Department of Neurosurgery, Massachusetts General Hospital, Harvard Medical School, Boston, MA 02115, USA.
[4]Ben May Department for Cancer Research, Pritzker School of Molecular Engineering, University of Chicago, Chicago, IL 60637, USA.
[5] National Institute for Locomotor Disabilities (Divyangjan), Kolkata 700090, India.
[6]State Key Laboratory of Resource Insects, Medical Research Institute, Southwest University, Chongqing 400715, China.
[7]Department of Pathology, Xiangya Hospital, Central South University, Changsha 410017, China.
[8]National Clinical Research Center for Geriatric Disorders, Xiangya Hospital, Central South University, Changsha 410017, China.
[9]Clinical Research Division, Department of Biosciences, School of Basic and Applied Sciences, Galgotias University, Greater Noida 203201, India.
[10]Preclinical Department, Universiti Kuala Lumpur, Royal College of Medicine Perak (UniKL-RCMP), Ipoh 30450, Malaysia.
[11]Mr. & Mrs. Ko Chi-Ming Centre for Parkinson's Disease Research, School of Chinese Medicine, Hong Kong Baptist University, Hong Kong SAR 999077, China.
[12]Department of Biochemistry, Karpagam Academy of Higher Education, Coimbatore 641021, India.
[13]Law Sau Fai Institute for Advancing Translational Medicine in Bone and Joint Diseases, School of Chinese Medicine, Hong Kong Baptist University, Hong Kong SAR 999077, China.
[14]Institute of Integrated Bioinfomedicine and Translational Science, School of Chinese Medicine, Hong Kong Baptist University, Hong Kong SAR 999077, China.
[15]Institute of Precision Medicine and Innovative Drug Discovery, HKBU Institute for Research and Continuing Education, Shenzhen 518000, China.
[*]Correspondence: yzhijun@hkbu.edu.hk; Tel.: +852-6769-4768 (ZY)

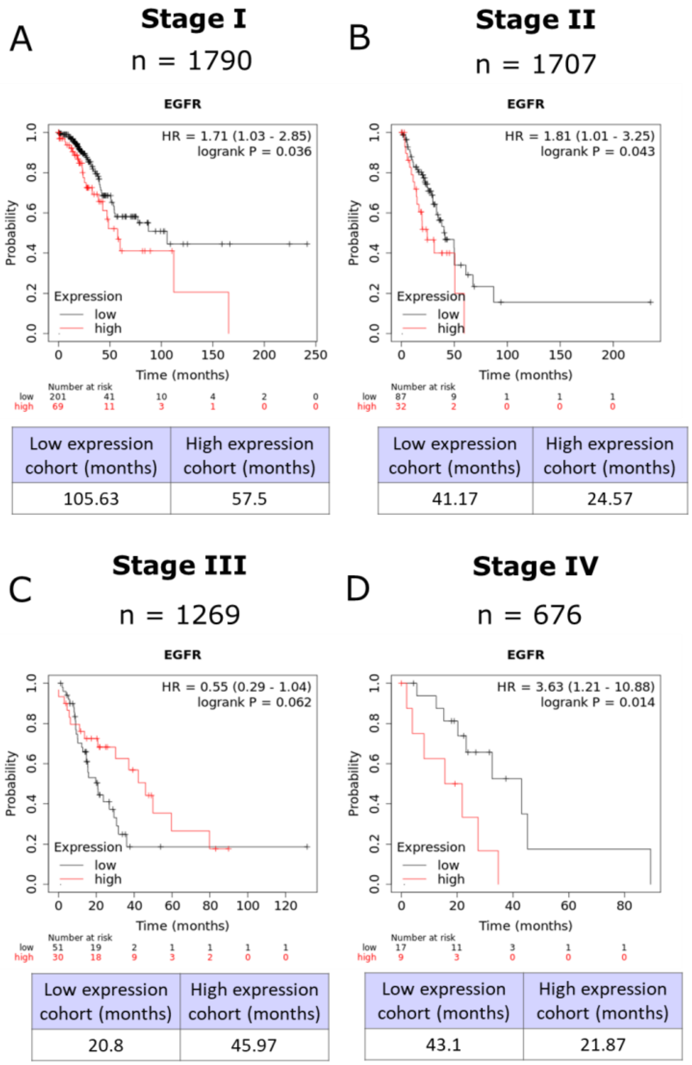

Supplementary Figure S1. Stage-dependent survival of lung cancer patients with the corresponding expression level of *EGFR*. Survival curves of patients with lung cancer with the expression of *EGFR* in **(A)** stage I, **(B)** stage II, **(C)** stage III, and **(D)** stage IV.

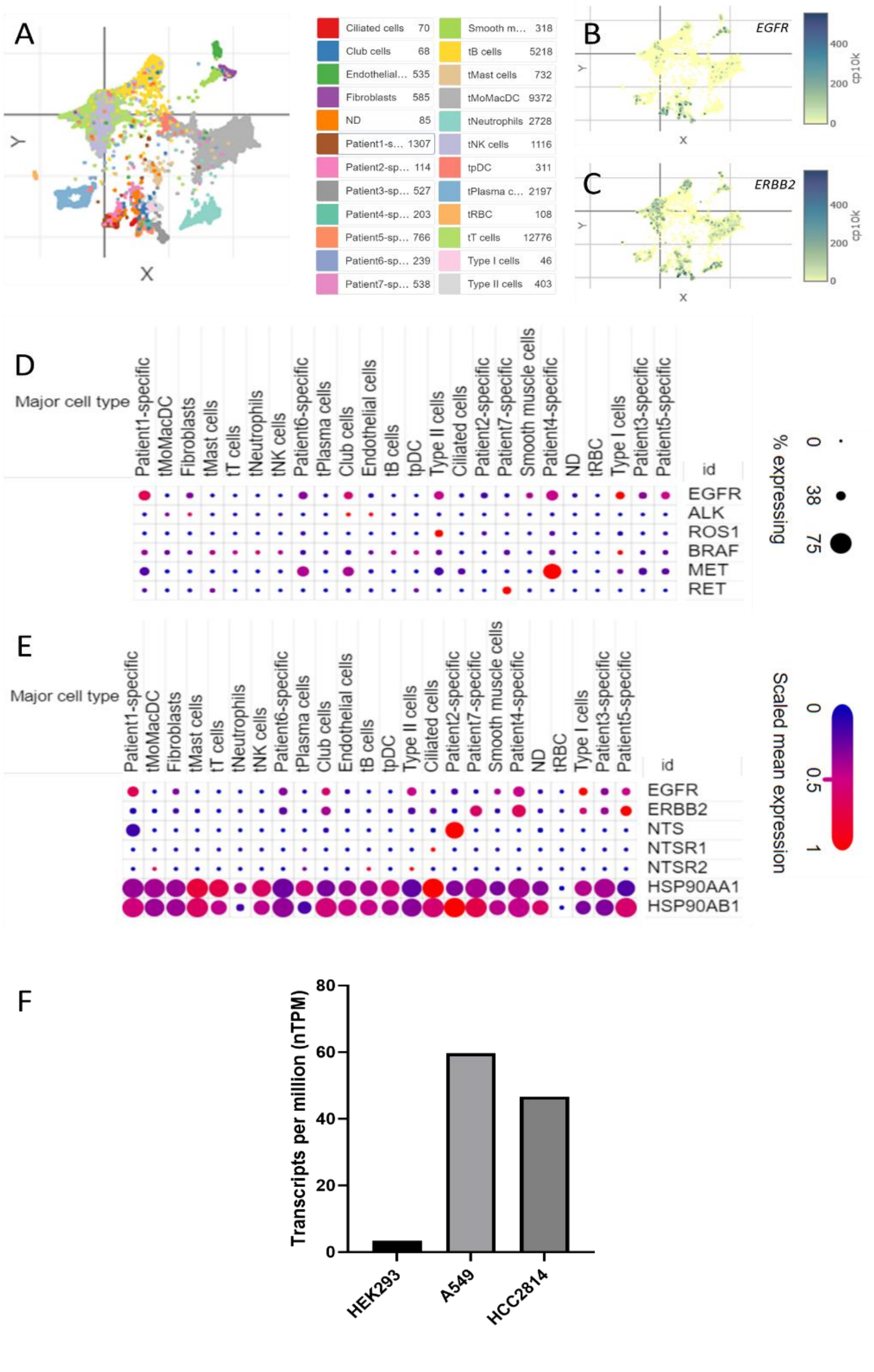

Supplementary Figure S2. Single-cell RNA-seq analysis of tumor microenvironments (TMEs) in lung cancer patients. (A) UMAP depicting the different cell types in the TME of human non-small cell lung cancer compared to control group (based on GEO: GSE127465). **(B, C)** UMAPs showing the expressions of (B) *EGFR* and (C) *ERBB2* in the clusters, representing cancer cell types. **(D, E)** Dot plots showing the cell type-specific expression of major cancer markers including *EGFR* and *ERBB2*. The figures are based on the analysis of single cell RNA-seq data publicly available (GSE127465). **(F)** Expression level of EGFR in HEK293, A549, and HCC2814 cell lines, as analyzed using protein atlas.

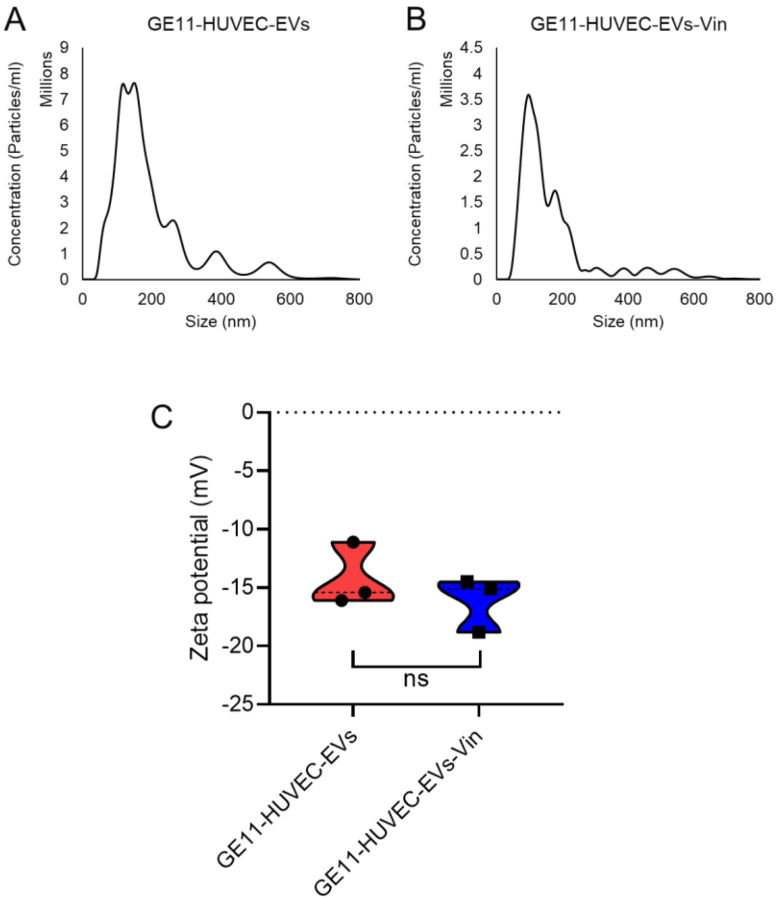

Supplementary Figure S3. Size distribution and zeta potential of GE11-HUVEC-EVs, without and with loading of vinorelbine. (A, B) The graph showing the size dependent concentration of GE11-HUVEC-EVs and GE11-HUVEC-EVs-Vin. (C) Violin plot showing the zeta potential of GE11-HUVEC-EVs and GE11-HUVEC-EVs-Vin.

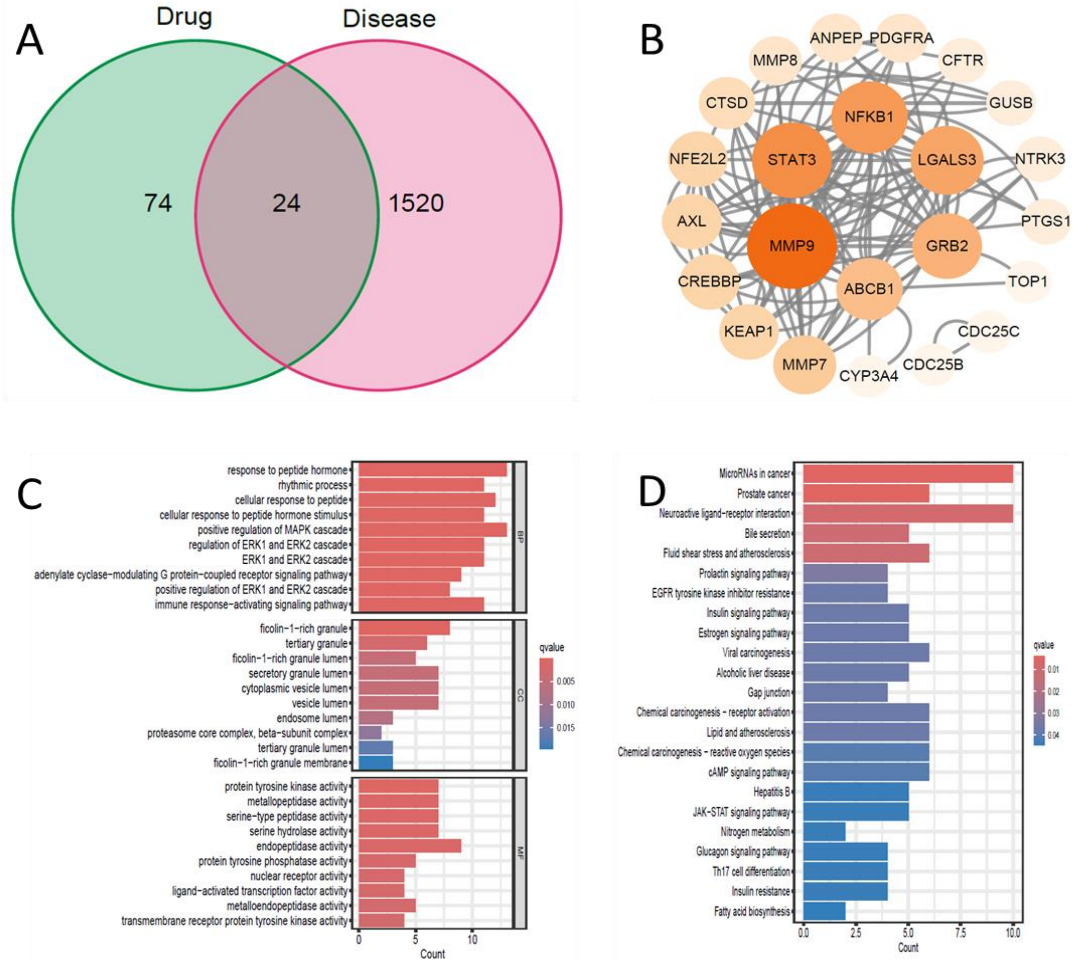

Supplementary Figure S4. Identification of potential lung cancer targets via *in silico* analysis. (A) The intersection of compound target proteins and lung cancer treatment targets resulted in 44 intersecting targets. **(B)** These 24 potential targets were imported into the STRING (https://cn.string-db.org/) online website to obtain a PPI network, which contains 24 nodes and 110 edges. The depth of color and circle size represent the corresponding size of the degree. **(C)** GO enrichment analysis was conducted on these 24 potential targets, and the top 10 enrichment results were displayed. **(D)** KEGG enrichment analysis was performed on these 24 potential targets, and the top 30 enrichment results were displayed.

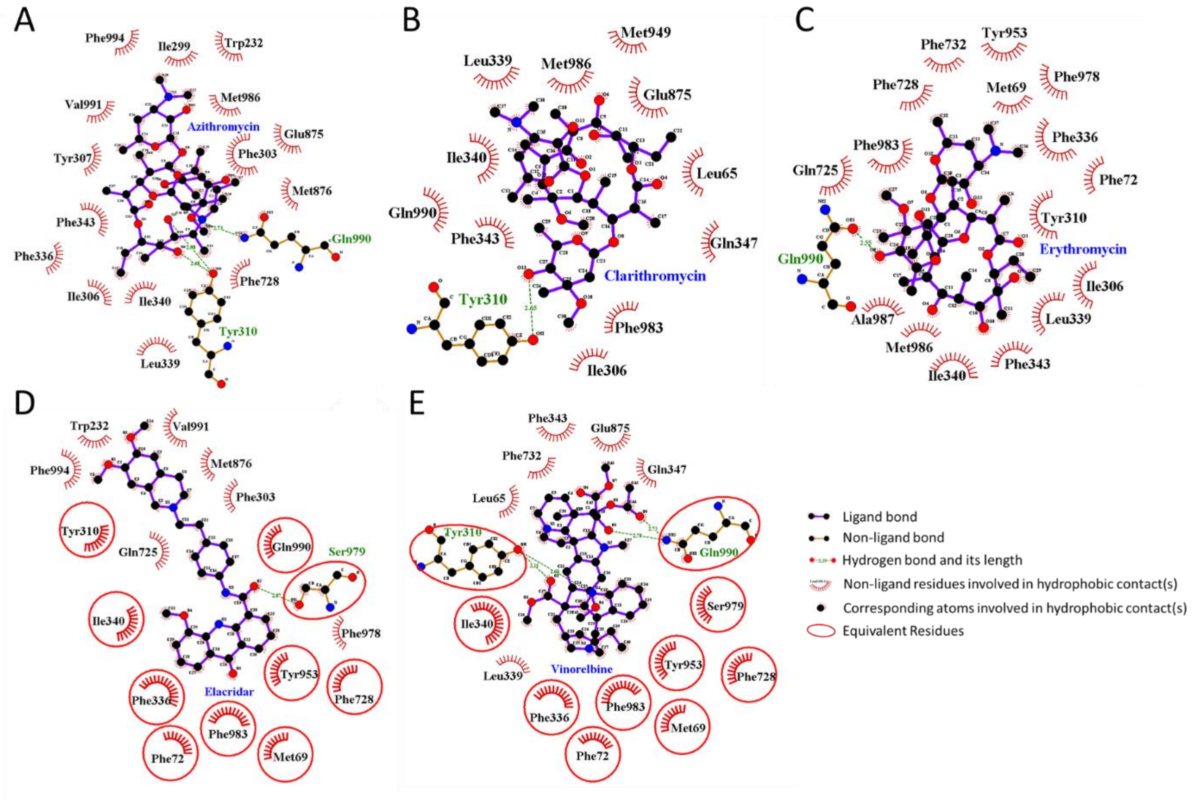

Supplementary Figure S5. Molecular docking of vinorelbine with ABCB1 protein. Depiction of H-bonding and hydrophobic interaction pattern of control drugs (azithromycin, clarithromycin, erythromycin, and elacridar) and vinorelbine with ABCB1.

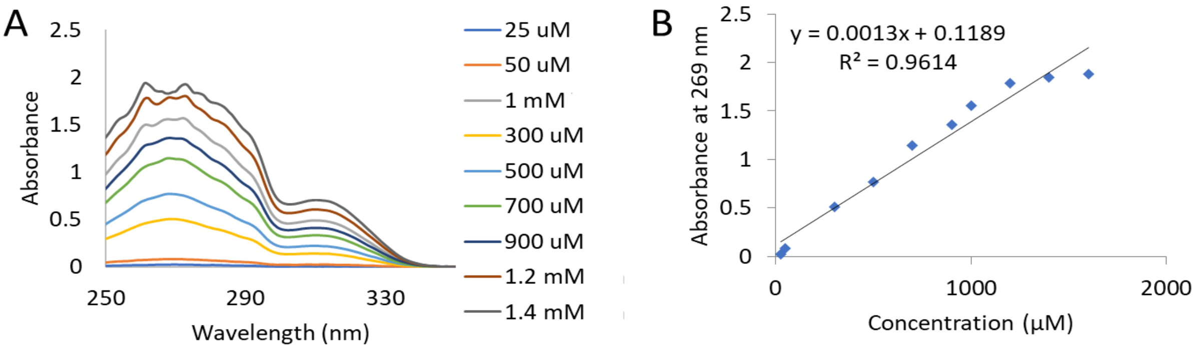

Supplementary Figure S6. Evaluation of vinorelbine encapsulation in GE11-HUVEC-EVs. (A) UV-visible spectra of difference concentrations of vinorelbine standard solutions. **(B)** Calibration standard graph showing the linear regression between concentration of vinorelbine standard solutions and the corresponding absorbance at the wavelength of 269 nm (λ_{max}).

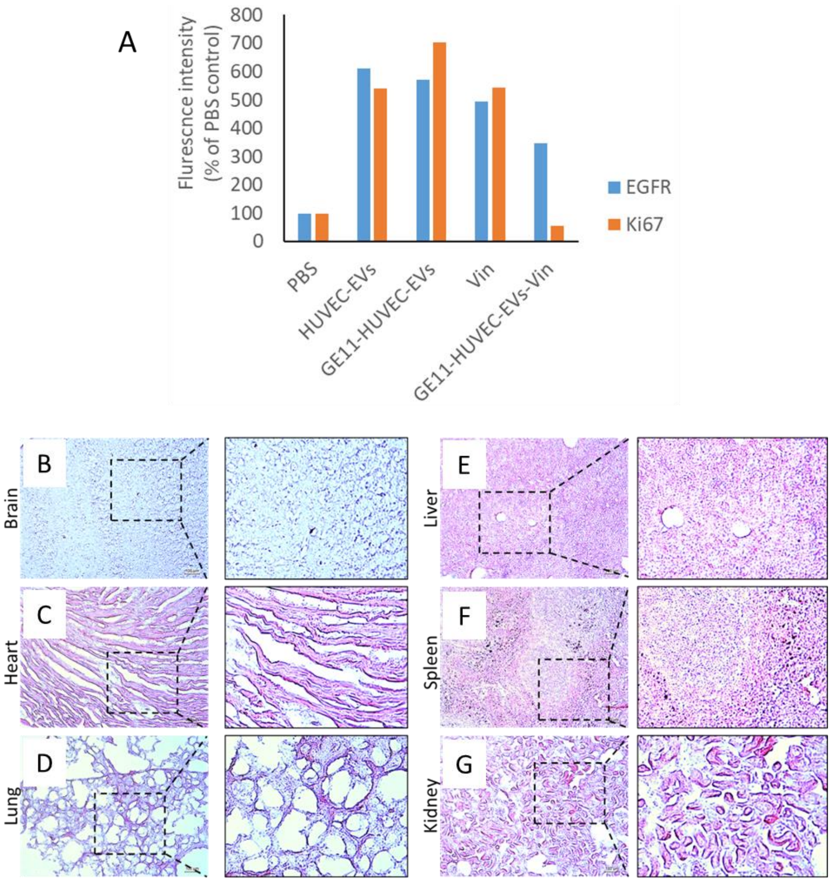

Supplementary Figure S7. Evaluation of in vivo toxicity of GE11-HUVEC-EVs in major organs of mice. (A) Bar graph showing the fluorescence intensity depicting the expression level of EGFR and Ki67. **(B-G)** H&E-stained images of major organs of lung cancer xenograft mice: (B) brain, (C) heart, (D) lung, (E) liver, (F) spleen, and (G) kidney.

Supplementary Table S1. Docking of different ligands with ABCB1

**Compound name**	**Binding Energy**	**Ki**	**H-bonding with ABCB1**
Azithromycin	-9.80 kcal/mol	65.09 nM	Gln990, Tyr310
Clarithromycin	-8.05 kcal/mol	1.25 uM	Tyr310
Erythromycin	-9.98 kcal/mol	48.71 nM	Gln990
Elacridar	-11.86 kcal/mol	2.03 nM	Ser979
Vinorelbine	-11.31 kcal/mol	5.09 nM	Tyr310, Gln990

Supplementary Table S2. Calculation of efficiency of vinorelbine encapsulation in GE11-HUVEC-EVs.

**Components**	**Absorbance**	**Conc. (μM)**
Initial drug	0.272	200.084615 (A)
Conditioned medium after centrifugation	0.187	134.7 (B)
Drug loading efficiency	= ((A-B)/A) x 100	32.6784194 %
